# Fibrin biopolymer as scaffold candidate to treat bone defects in rats

**DOI:** 10.1590/1678-9199-JVATITD-2019-0027

**Published:** 2019-11-04

**Authors:** Claudia Vilalva Cassaro, Luis Antonio Justulin, Patrícia Rodrigues de Lima, Marjorie de Assis Golim, Natália Perussi Biscola, Mateus Vidigal de Castro, Alexandre Leite Rodrigues de Oliveira, Danuta Pulz Doiche, Elenize Jamas Pereira, Rui Seabra Ferreira, Benedito Barraviera

**Affiliations:** 1 Center for the Study of Venoms and Venomous Animals (CEVAP), São Paulo State University (UNESP), Botucatu, SP, Brazil.; 2 Botucatu Medical School (FMB), São Paulo State University (UNESP), Botucatu, SP, Brazil.; 3 Extracellular Matrix Laboratory, Botucatu Biosciences Institute (IBB), São Paulo State University (UNESP), Botucatu, SP, Brazil.; 4 Flow Cytometry Laboratory, Blood Center, Botucatu Medical School (FMB), São Paulo State University (UNESP), Botucatu, SP, Brazil.; 5 Department of Structural and Functional Biology, Biosciences Institute (IB), University of Campinas (UNICAMP), Campinas, SP, Brazil.; 6 Department of Animal Reproduction and Veterinary Radiology, School of Veterinary Medicine and Animal Husbandry, São Paulo State University (UNESP), Botucatu, SP, Brazil.

**Keywords:** Bone regeneration, Biomaterials, Fibrin sealant, Fibrin biopolymer, Biphasic calcium phosphate, Mesenchymal stem cells

## Abstract

**Background:**

Bone tissue repair remains a challenge in tissue engineering. Currently, new materials are being applied and often integrated with live cells and biological scaffolds. The fibrin biopolymer (FBP) proposed in this study has hemostatic, sealant, adhesive, scaffolding and drug-delivery properties. The regenerative potential of an association of FBP, biphasic calcium phosphate (BCP) and mesenchymal stem cells (MSCs) was evaluated in defects of rat femurs.

**Methods:**

Adult male Wistar rats were submitted to a 5-mm defect in the femur. This was filled with the following materials and/or associations: BPC; FBP and BCP; FBP and MSCs; and BCP, FBP and MSCs. Bone defect without filling was defined as the control group. Thirty and sixty days after the procedure, animals were euthanatized and subjected to computed tomography, scanning electron microscopy and qualitative and quantitative histological analysis.

**Results:**

It was shown that FBP is a suitable scaffold for bone defects due to the formation of a stable clot that facilitates the handling and optimizes the surgical procedures, allowing also cell adhesion and proliferation. The association between the materials was biocompatible. Progressive deposition of bone matrix was higher in the group treated with FBP and MSCs. Differentiation of mesenchymal stem cells into osteogenic lineage was not necessary to stimulate bone formation.

**Conclusions:**

FBP proved to be an excellent scaffold candidate for bone repair therapies due to application ease and biocompatibility with synthetic calcium-based materials. The satisfactory results obtained by the association of FBP with MSCs may provide a more effective and less costly new approach for bone tissue engineering.

## Background

Regeneration of bone tissue starts immediately after its damage, and involves osteoinduction and osteoconduction, two phenomena that result in the reestablishment of the structure and function [1]. Throughout life, under normal conditions, this tissue is constantly renewed [2,3]. However, several factors may impair the repair process, such as extensive defects, trauma, infection, tumors, bone abnormalities and congenital malformations. The occurrence of non-unions require the use of grafts or implants that act as support and stimulate the process of tissue restoration. Thus, tissue engineering approaches aim to develop regenerative therapies for bone tissue in order to restore its shape and function by combining cells, signaling molecules and biocompatible materials, preferably associated with a bioactive scaffold [4].

Currently, autogenous bone grafts are still considered the gold standard in bone grafting due to their osteogenic properties [1]. However, their application presents drawbacks such as the limited amount of donor bone tissue and the need for a second surgery to remove the graft, causing an increase in recovery time. In this scenario, synthetic bone grafts offer an important alternative due to their unlimited availability [5]. Compounds containing calcium phosphates comprise the main class of biomaterials used in the replacement and in the regeneration of bone tissue. Their main characteristics include biocompatibility, bioactivity and absence of toxicity and immunogenicity. In addition, these materials are excellent osteoconductors and increase mechanical resistance at the defect site [6,7]. Among these materials are hydroxyapatite (HA), β-tricalcium phosphate (TCP) and a combination of the two known as biphasic calcium phosphates (BCP) [8]. This latter compound is widely used due to its additional advantage of controlling the level of degradation by adjusting the proportion between the two components [7].

The association between calcium phosphate materials and mesenchymal stem cells presents good osteogenic potential, since these stem cells have an appropriate surface for cellular adhesion and activity, thus acting as a stimulus for the differentiation in osteoblasts [9-13]. Moreover, the unique ability of mesenchymal cells to promote bone healing, combined with their ease of isolation, provides a more appropriate therapy for patients who suffer from limitations in the regeneration of this tissue [14,15]. However, for satisfactory cell action, adequate anchoring at the application site is imperative, and may be provided by a biological scaffold. Three-dimensional scaffolds provide a suitable environment for tissue regeneration, acting as a framework for bone formation and are easily implanted with cells, biomaterials or growth factors, thus providing structural and environmental stability for cell and tissue regeneration [16-19].

Fibrin sealants are natural biopolymers endowed with adhesive, sealant, and hemostatic properties, and are suitable for cell adhesion and tissue stability. They are widely used as scaffolds and in drug delivery systems, enabling the precise application of biomaterials, cells, enzymes or growth factors [20-22]. Traditional commercial fibrin sealants have been produced from thrombin obtained from human or bovine blood, and human fibrinogen [22,23]. This biomaterial, under exceptional conditions, can transmit bloodborne diseases [23,24]. Therefore, researchers from the Center for the Study of Venoms and Venomous Animals (CEVAP) at São Paulo State University (UNESP), Brazil, proposed the production of a new fibrin biopolymer (FBP), previously identified as heterologous fibrin sealant, which does not use human blood derived products. Thus, a thrombin-like serine protease enzyme isolated from *Crotalus durissus terrificus* snake venom, and a cryoprecipitate rich in fibrinogen extracted from *Bubalus bubalis* were employed [25]. This product was patented, tested through several animal experiments and approved by the Brazilian Health Regulatory Agency (ANVISA) for phase I/II clinical trials in the treatment of patients with chronic venous ulcers, in which the product was proven safe [25-45].

It was shown that the fibrin biopolymer produced by CEVAP has an excellent interaction with the mesenchymal stem cells derived from the bone marrow of rats [30]. It forms a three-dimensional structure that enables cell adhesion, differentiation and proliferation, properties also observed in commercial polymers [46,47]. In addition, satisfactory results were obtained by associating FBP with synthetic hydroxyapatite in the treatment of cranial defects in rats [43]. Recently, an association between FBP and mesenchymal stem cells in femoral defects of osteoporotic rats was also validated [45].

Therefore, the present study aimed to evaluate for the first time the repair potential of calcium phosphate biomaterial applied together with FBP associated or not with mesenchymal stem cells in rats submitted to a femoral bone defect.

## Methods

## Animals

Eighty male Wistar rats (*Rattus norvegicus*) weighing an average of 350 ± 20 g were randomly divided into five experimental groups, while ten rats aged 21 days were submitted to bone marrow harvest to achieve mesenchymal stem cell expansion. The rats were provided by the animal house of the Laboratory of Experimental Medicine, UNESP, Botucatu. All animals were kept at 21 ± 2°C under a 12-hour light/dark cycle, with access to food and water *ad libitum*. The present study was approved by the Animal Ethics Committee of Botucatu Medical School, São Paulo State University (UNESP - protocol number 1164/2016).

## Fibrin Biopolymer

**Figure 1.**
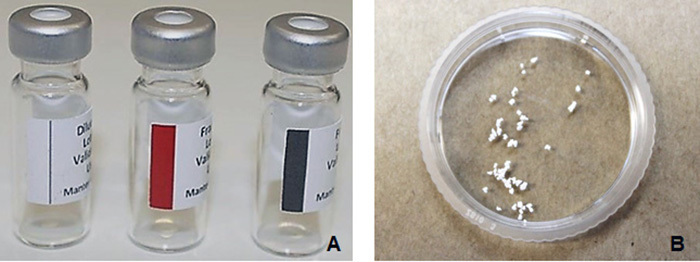
**(A)** Fibrin biopolymer vials [26]. **(B)** Presentation of the biphasic calcium phosphate: aspect of the granules.

The fibrin biopolymer (FBP) (Fig. 1A) was supplied by CEVAP. The components and formula of the product are covered by patents (registry numbers: BR1020140114327 and BR1020140114360). The components were mixed upon use as follows: 10 μL of fraction 1 (gyroxin), 25 μL of fraction 2 (fibrinogen cryoprecipitate) and 15 μL of fraction 3 (diluent); resulting in a final volume of 50 μL of sealant and maintaining the standard concentration.

## Biphasic Calcium Phosphate Biomaterial

The biphasic calcium phosphate (BCP) (Graftys^^®^^ BCP, Graftys Sarls, France, Anvisa n. 80517190002) (Fig. 1B) was kindly supplied by LAS Brasil importer. The synthetic material was presented in granules varying between 0.5 and 1 mm and consisted of 60% of hydroxyapatite and 40% of β-tricalcium phosphate. Its porosity varied between ≤ 10 μm and 100 μm [48]. Fifteen milligrams of previously aliquoted and stored material was implanted at the site of interest.

## Mesenchymal Stem Cells

### *Isolation and Expansion*

In order to harvest the cells from bone marrow, rats were euthanized by isoflurane overdose at minimum alveolar concentration (MAC > 5%) and, after the unconsciousness of the animals was confirmed, cervical dislocation. Bone marrow cells were obtained by centrifugation of the femurs and tibias, which were vertically positioned in conical tubes containing 1000 μL of Dulbecco’s Modified Eagle Medium (DMEM^^®^^, Gibco-Invitrogen, USA) high glucose, and centrifuged at 2,500 rpm for four minutes. The bones were then discarded and the precipitate was resuspended.

The obtained solution was transferred to 25 cm^2^ culture flasks containing 4 mL of DMEM^^®^^ (supplemented with 20% fetal bovine serum, 100 μg/mL penicillin/streptomycin and 3 μg/mL amphotericin B), comprising a total volume of 5 mL. The flasks were kept in an incubator with a 5% CO_2_ atmosphere at 37.5°C. The culture medium was replaced every 3 to 5 days, depending on the growth of cells and adherence on the bottle surface, both monitored by light microscopy.

When cells reached about 90% confluence, the cell passage was performed. For this process, the culture medium in the flask was discarded; 2 mL of phosphate-buffered saline (PBS) was added for washing and discarded thereafter. After washing, 2 mL of trypsin was added and the flask was maintained in the incubator for five minutes. With the cells in suspension, 2 mL of supplemented DMEM^^®^^ was added. The solution was then transferred to 15-mL conical tubes and centrifuged at 1700 rpm for ten minutes. After centrifugation, the supernatant was discarded and the pellet containing the cells was resuspended in 1000 μL of DMEM^^®^^. The solution was then transferred to flasks of the appropriate size.

### Characterization

Cultivated mesenchymal stem cells (MSCs) on the fourth passage were characterized by flow cytometry (BD FACSCalibur™, BD Biosciences)FACS Calibur^®^; BD Pharmingen) using the positive surface markers CD90, CD44 and ICAM-1, and negative expression markers CD45, CD11b, MHC class I and RT1-aw2 [49]. Specifications of each marker are presented in Additional file 1. The preparation consisted of an adjustment in cell volume to 2 × 10^5^ cells per tube/marker. Antibodies were added according to specific dilution also described in Additional file 1. The samples were then incubated at room temperature for one hour and washed with PBS [45]. 

## Association of FBP with Mesenchymal Stem Cells and Calcium Phosphate

At the bone defect site and depending on the group, 50 μL of FBP, 15 mg of biphasic calcium phosphate, and 10 μL of complete culture medium containing an average of 3 × 10^5^ cells previously characterized were added. In the group in which only the biphasic calcium phosphate (G2) was implanted, the granules were placed directly at the lesion/implant receptor site, adding and compacting the granules with the help of a spatula. For the association of BCP and FBP (G3), the fractions 2 and 3 of the latter were homogenized on a cell culture plate, the calcium phosphate was added and, finally, fraction 1 was added. The clot formed was then transferred to the defect site.

After counting the mesenchymal stem cells, and associating them with FBP only (G4) or FBP and with BCP (G5), a 2-mL volume was transferred to microtubes and centrifuged. Next, the cells were resuspended in 2-mL microtubes, comprising a complete medium volume of 10 μL, and then added to a microtube containing the calcium-based biomaterial and the FBP fractions as previously described. The formed clot was then transferred to the lesion site. In the control group, no material was received.

## ***In Vivo* Experimental Procedures**

*Experimental Design*

A 5-mm bone defect was performed on the right femur of each rat, described as follows. Animals were divided into five experimental groups of eight individuals each, namely:

- Group 1 (G1): bone defect without material filling (control group).
- Group 2 (G2): bone defect filled with BCP.
- Group 3 (G3): bone defect filled with FBP + BCP.
- Group 4 (G4): bone defect filled with FBP + MSCs.
- Group 5 (G5): bone defect filled with FBP + BCP + MSC.

*Femur-Defect Surgical Procedure*

Animals were anesthetized with isoflurane at MAC of 3% for induction and 1.5% for maintenance, without need for mechanical ventilation. After confirmation of anesthetic induction, the animals were placed in lateral decubitus position and trichotomy was performed on the anterior region of the right thigh. A medial longitudinal incision was made on the skin of the anterior distal third of the region. The skin was laterally removed, and then the femoral quadriceps muscle was separated exposing the periosteum, which was sectioned transversely and disjointed from the bone surface.

**Figure 2.**
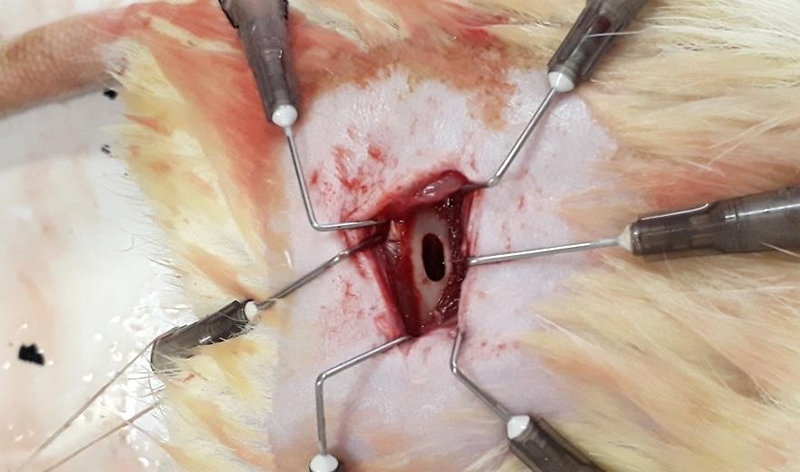
Femoral 5-mm bone defect site.

After exposure of the bone tissue, a bone defect of 5 mm diameter and 2 mm width was induced using a drill bit coupled to a dental micromotor (Beltec^®^ model LB100; Brazil) at about 3000 rpm, thus promoting a bone defect in the proximal third of the right femur diaphysis (Fig. 2). Throughout the defect induction procedure, the lesion site was irrigated continuously with physiological solution in order to avoid overheating in the region. According to the group, the mixture of components was added to the promoted bone defect. After stabilizing the graft, suturing was performed. In the postoperative period, the animals received food and water *ad libitum* and were not immobilized at any time. The rats received postoperative analgesia consisting of tramadol hydrochloride at 5 mg/kg, ketoprofen at 5 mg/kg (Ketofen^®^ 10%) and enrofloxacin hydrochloride at 8 mg/kg (Chemitril^^®^^ 2.5%) subcutaneously at 12-hour intervals for three days [51 ].

*Euthanasia*

Animals were euthanized by isoflurane overdose (MAC > 5%) and, after the unconsciousness of the animals was confirmed, cervical dislocation. The procedure occurred in two periods for observation and analysis of samples: at 30 and 60 days after surgery, four animals from each group were euthanized. In these two periods, before euthanasia, the region of interest was scanned by computed tomography. The collected bones were then forwarded for histological and for scanning electron microscopy analysis. The macroscopic aspect of the femurs and surrounding areas at the moment of euthanasia was also considered.

## Computed Tomography Analysis

At 30 and 60 days after the surgical procedure, the animals were submitted to computed tomography (CT) scan in order to evaluate the repair process at the lesion site. The procedure was carried out by a helical single-channel tomograph (Shimadzu^®^ SCT-7800 TC, Japan), whose additional specifications are displayed in Additional file 2. For the scan, animals were previously anesthetized with ketamine and xylazine hydrochloride at the dose of 0.10 mL / 100 grams of body mass and positioned in dorsal decubitus. The images obtained were studied in the cross-sectional, longitudinal and coronal sections, whereas the tomographic-graphical appearance of the implants was evaluated considering adjacent opacification, Hounsfield Unit values, bone proliferation, consolidation and remodeling processes in the region of interest [52].

## Scanning Electron Microscopy (SEM) Analysis

After collection, the femurs were initially fixed in Karnovsky’s solution for 36 hours. After fixation, samples were washed in phosphate buffer and immersed in 0.5% osmium tetroxide solution for 60 minutes and then dried to the critical point, positioned over stubs and coated with gold (sputtering process). The samples were analyzed using a Jeol^^®^^ JSM 5800LV (USA) microscope at 10 kV.

## Histological Analysis

The femurs were collected and fixed in 10% formaldehyde solution for 24 hours. Subsequently, the material was subjected to decalcification in 30% formic acid solution for 15 days. After decalcification, the bones were reduced to the region of interest, fixed in 70% alcohol for 12 hours, dehydrated in an increasing series of ethanol, diaphanized in xylol and, finally, embedded in paraffin. Semi-serial longitudinal 5-μm sections of the bone tissue were obtained and stained with hematoxylin and eosin (HE). The slides obtained were visualized and photographed (Leica DM500^^®^^ microscope, Leica DMC2900^^®^^ camera; Germany) with aid of the equipment described in Additional file 3 at magnifications of 4x, 10x and 20x, to evaluate morphological characteristics. Stereological analysis was performed based on images obtained by 10x magnification (Zeiss Stemi 2000^®^ magnifier, Germany; Dino Eye AM7025X^^®^^, Taiwan) according to the methodology described by Weibel et al. [53] in order to quantify formed bone, biomaterial, bone marrow and cellularized connective tissue on lesion area. 

## Statistical Analysis

Based on the results of the stereological analysis, the groups were compared by ANOVA, followed by Tukey’s test for multiple comparisons. Differences were considered statistically significant if p < 0.05. The data analysis was performed using the software SPSS version 21.0.

## Results

## Mesenchymal Stem Cell Characterization

**Figure 3.**
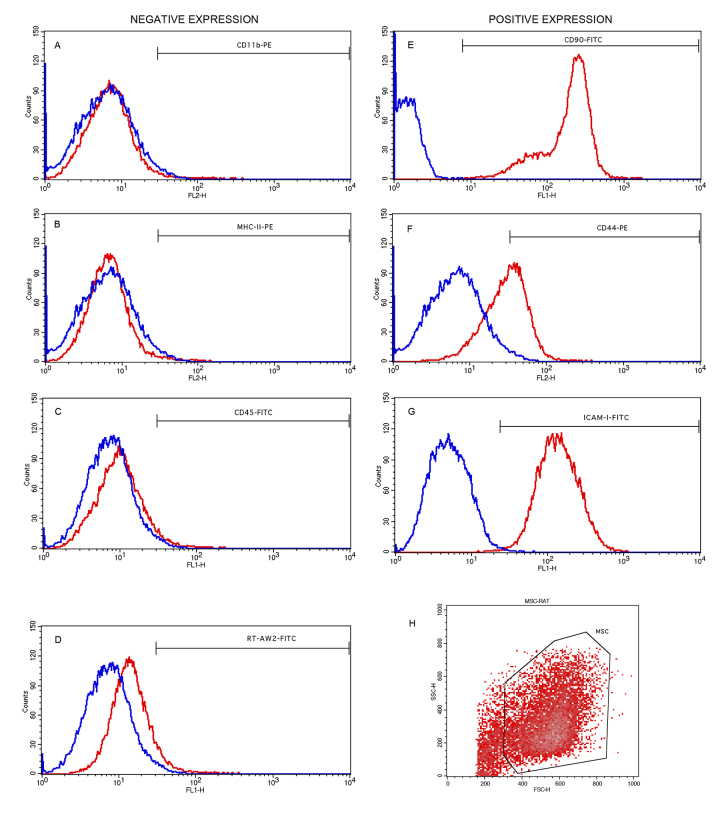
Flow cytometry analysis of mesenchymal stem cells characterized by surface markers. Blue line: negative markers; red line: positive markers. **(A)** CD11B, **(B)** MHC class II, **(C)** CD45, **(D)** RT1-Aw2, **(E)** CD90, **(F)** CD44, **(G)** ICAM1, and **(H)** gate of analyzed cell population.

The applied mesenchymal stem cells presented the expressions represented in histograms shown in Figure 3 and were positive for the markers CD90 (98.29%), CD44 (63.21%) and ICAM1 (99.3%), and negative for CD45 (6.57%), CD11b (4.35%), RT1-aw2 (9.4%) and MHC Class II (3.54%), in agreement with the literature [49,54].

## Surgical Procedure and Euthanasia

**Figure 4.**
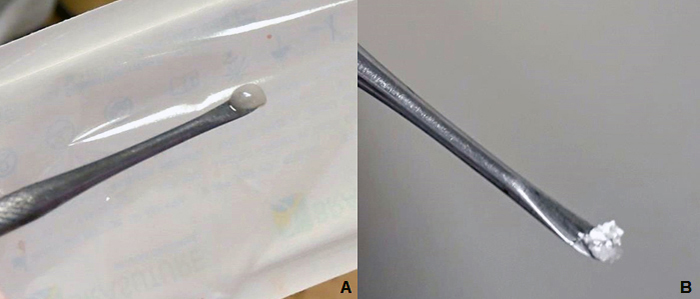
Stable clot formed by fibrin biopolymer containing **(A)** mesenchymal stem cells and **(B)** biphasic calcium phosphate.

**Figure 5.**
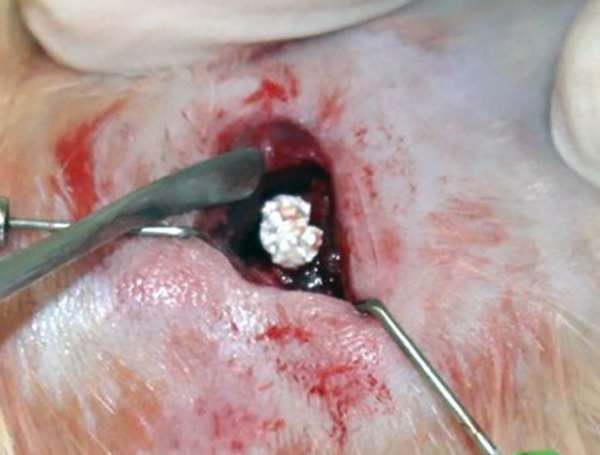
Implantation of the composite at the bone defect site.

At the time of surgery, the group presenting the easiest application of materials was FBP, given that the clot formed by FBP contained biphasic calcium phosphate while its mesenchymal stem cells (Fig. 4) were much easier to handle, mold and accommodate at the injury site (Fig. 5).

After the surgery, the incision site showed no signs of inflammation and animals presented normal locomotion. At the time of euthanasia, no inflammatory infiltrates or rejection at the graft site was observed.

## CT Analysis

**Figure 6.**
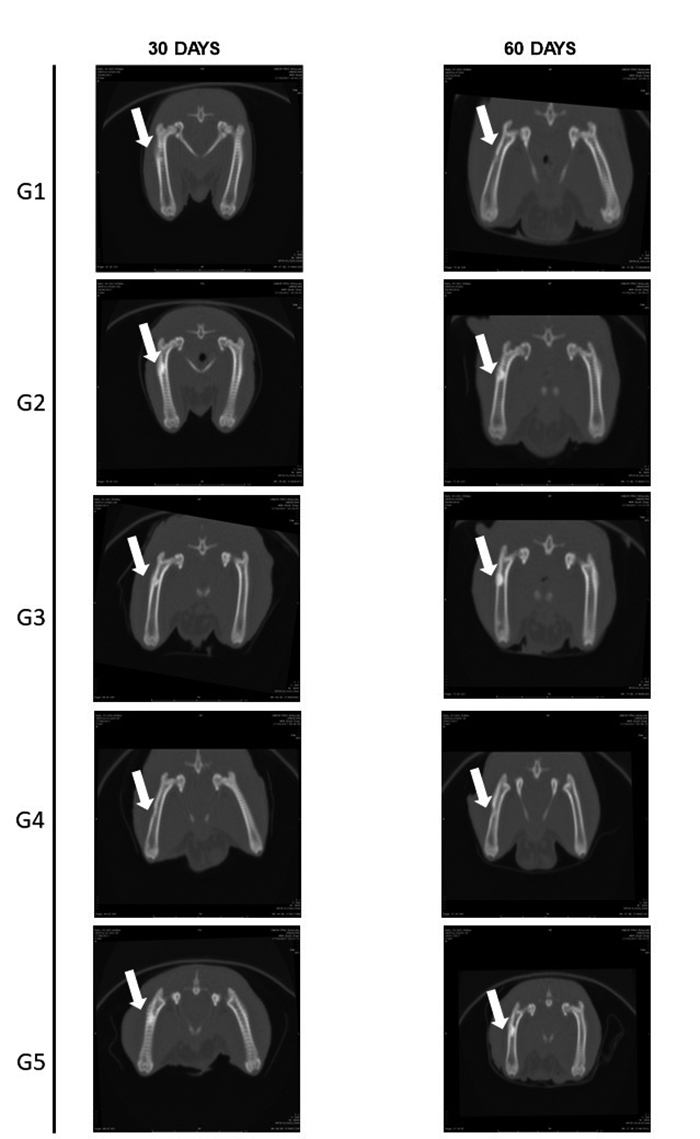
Representative images on coronal plane of the right femur of rats submitted to bone defect (white arrows) at 30 and 60 days after surgical procedure by CT scan.

**Figure 7.**
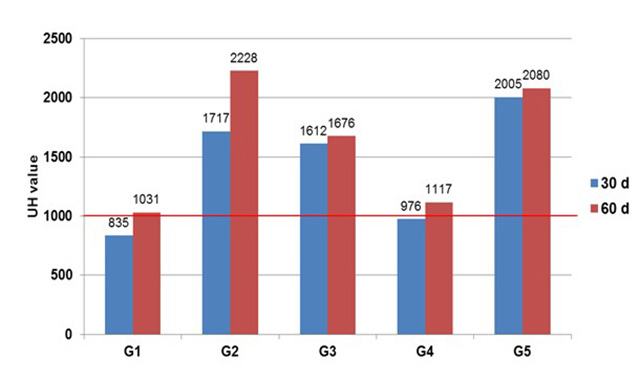
Mean Hounsfield unit (HU) value at the defect site of all groups 30 and 60 days after surgical procedure. Observation by CT scan. Red line indicates minimum value expected for regular cortical bone.

Through the images obtained by tomography (Fig. 6), a lesion site was visible in all groups and periods. The quantitative parameter evaluated was the HU value (Hounsfield units) (Fig. 7), a measure related to the density of the tissue in formation, which in normal cortical bone tissue has a value of 1000 HU or more [52]. At 30 days, the mean value per group was less than 1000 only in the control group. At 60 days, the HU value was higher than 1000 in all groups, but the control still presented the lowest value among all groups.

According to the qualitative parameters considered, proliferation - which indicates bone activity at the lesion site - suggested that the total consolidation had not yet occurred at day 60. In addition, remodeling in the region was also active at 60 days, but the control group showed cortical misalignment and separation between the edges of the lesion (Table 1). Opacification, which indicates the similarity of the radiopacity area of the lesion within adjacent intact tissue, was greater in the groups in which biphasic calcium phosphate was applied. Detailed information about the parameters evaluated is contained in Additional file 4.

**Table 1.** Qualitative parameters for bone growth and consolidation by CT scan.

	Group 1		Group 2		Group 3		Group 4		Group 5	
	30 days	60 days	30 days	60 days	30 days	60 days	30 days	60 days	30 days	60 days
Proliferation	-	-+	+	+	+	+	+	++	+	+
Remodeling	-+	-+	+	+	+	+	++	++	+	++
Opacification	-+	-+	++	++	++	++	+	+	++	++

-: absent; -+: minor significance; +: limited significance; ++: significant.

## Macroscopic Aspect and SEM Analysis

**Figure 8.**
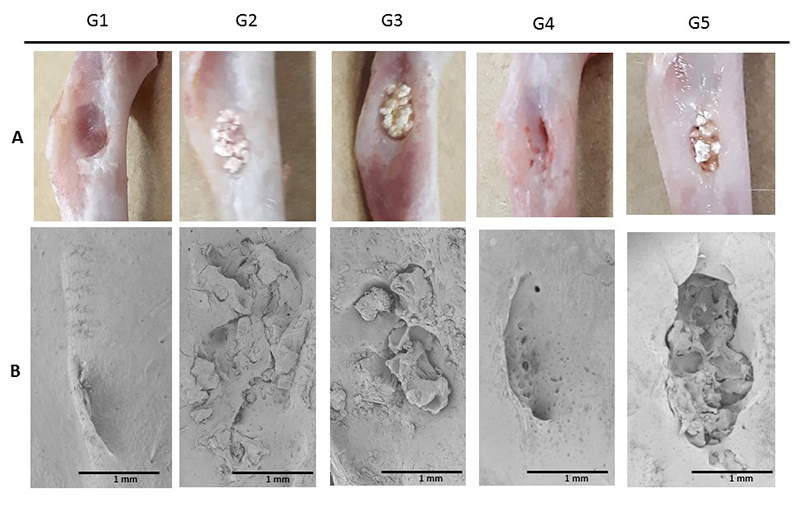
Representative images of the region of interest: **(A)** macroscopic and **(B)** by scanning electron microscopy view, 30 days after surgical procedure.

**Figure 9.**
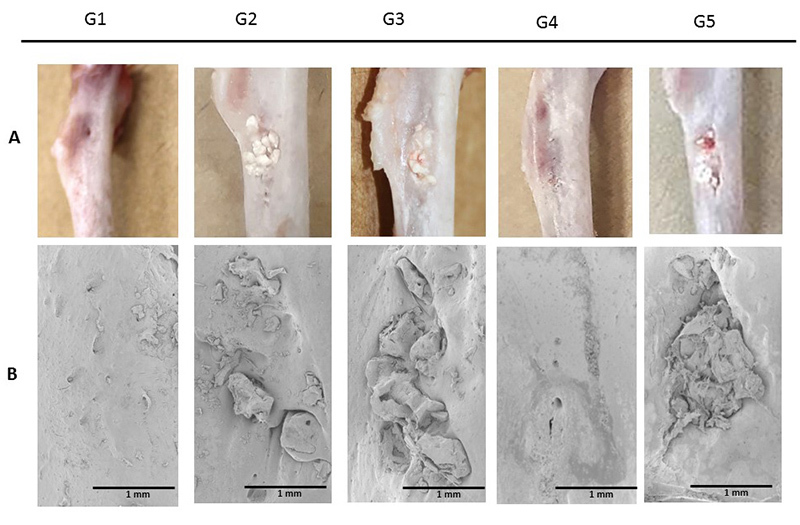
Representative images of the region of interest: **(A)** macroscopic and **(B)** by scanning electron microscopy view, 60 days after surgical procedure.

Figures 8 and 9 show the general aspect of the lesion region at 30 and 60 days after the surgical procedure, at the moment in which material was collected after euthanasia and subjected to scanning electron microscopy (SEM). Macroscopically and at 30 days, the edges of the lesion were distinguishable in all experimental groups, with a significant approximation of the edges of the lesion in Group 4. Through microscopic images, groups 1 and 4 (both without biphasic calcium phosphate material) showed uniform coverage at the lesion site, while the groups treated with calcium phosphate presented the characteristic morphology of the granules. 

At 60 days, the approximated edges of the lesion were visible in all the groups, with integration of the lesion edges into the applied association, but without apparent resorption in the central region in the groups that received the biomaterial. The images obtained through the scan corroborate the approximation of the lesion margins and did not enable visualization of the lesions. The control group and the group receiving association of fibrin biopolymer and cells (Group 4) presented total superficial coverage in the lesion area. Yet again, calcium phosphate remained without great resorption in the central region of the lesion.

## Histological Analysis

**Figure 10.**
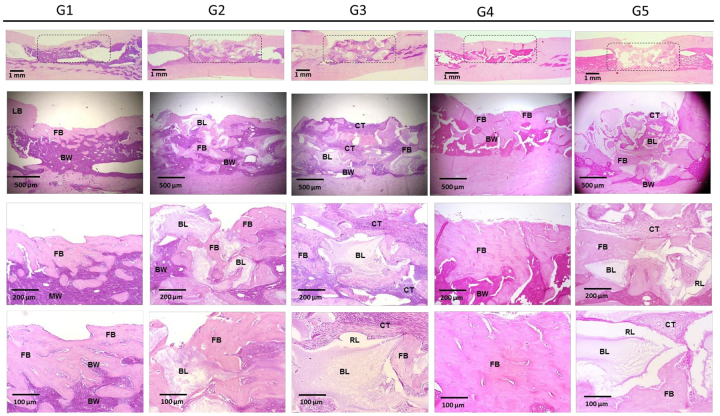
Representative images of histological sections stained with hematoxylin and eosin from the region of interest at 30 days after surgical procedure. LB: adjacent lamellar bone; FB: newly formed bone; BL: calcium phosphate biomaterial; RL: resorption lacunae; BW: bone marrow; CT: cellularized connective tissue.

**Figure 11.**
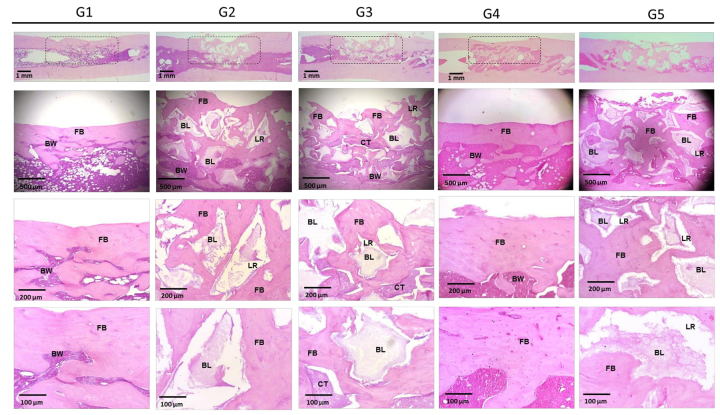
Representative images of histological sections stained with hematoxylin and eosin from the region of interest at 60 days after surgical procedure. LB: adjacent lamellar bone; FB: newly formed bone; BL: calcium phosphate biomaterial; RL: resorption lacunae; BW: bone marrow; CT: cellularized connective tissue.

Figures 10 and 11 present the images of the histological sections stained with hematoxylin and eosin from the defect region 30 and 60 days after implantation. Progressive deposition of bone matrix was detectable in all groups. Groups 2, 3 and 5 showed integration of the calcium phosphate biomaterial into the adjacent newly formed matrix, with a large presence of resorption lacunae, which were less numerous in Group 2. In Group 4, the bone formation was very copious, linear and organized, with few bone marrow infiltrates. In groups 2 and 5 and at 30 days, a greater presence of highly cellularized connective tissue was observed. 

**Figure 12.**
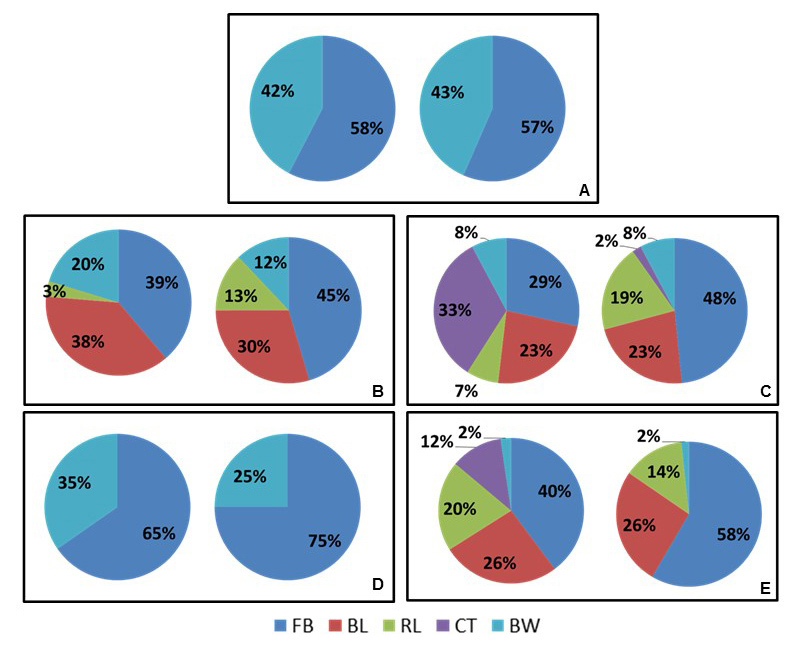
Relative quantification of the defect site of all groups 30 and 60 days after procedure observed in histological sections. **(A)** Group 1; **(B)** Group 2; **(C)** Groups 3; **(D)** Group 4; **(E)** Group 5. FB: newly formed bone; BL: calcium phosphate material; RL: resorption lacunae; BW: bone marrow; CT: cellularized connective tissue.

**Figure 13.**
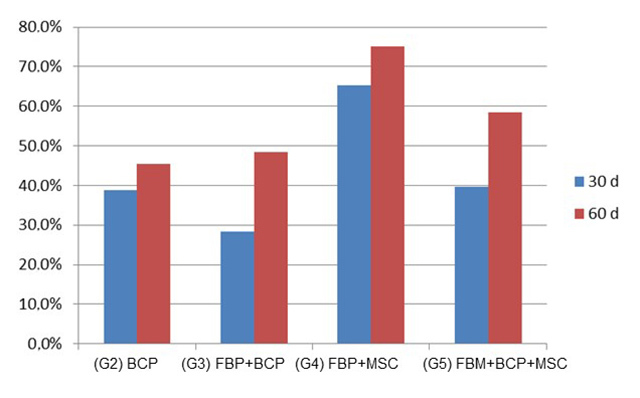
Relative quantification of progression of bone formation of treated groups 30 and 60 days after procedure observed in histological sections.

At 60 days, the depositions of bone matrix were similar in groups 2, 3, and 5, and more evident in Group 4, which received the association of fibrin biopolymer and mesenchymal stem cells. The bone formation in the treated groups, quantified by stereological analysis (Figs.12 and 13; Additional file 3), can be represented by: FBP + MSC > FBP + BCP + MSC > FBP + BCP > BCP. Statistical analysis showed significantly higher bone formation in Group 4 (Table 2; Additional file 4).

**Table 2.** Statistical comparison among groups by ANOVA test. Data presented by mean and standard deviation.

Variable	G1 (control)	G2 (BCP)	G3 (FBP + BCP)	G4 (FBP + MSC)	G5 (FBP + BCP + MSC)	*p value*
FB30*	56.5 ± 5.6	38.7 ± 4.6	28.4 ± 5.1	65.3 ± 4.1	39.7 ± 15.3	< 0.001
BW30	43.4 ± 5.6	20.2 ± 10.7	7.8 ± 3.6	34.6 ± 4.1	2.3 ± 2.8	< 0.001
FB60	57.6 ± 11.3	45.4 ± 12.2	48.4 ± 5.7	75 ± 2.7	58.3 ± 7.8	0.002
BW60	42.3 ± 11.3	12 ± 5.8	7.7 ± 1.9	25 ± 2.7	1.6 ± 1.9	< 0.001
DifFB	1.2 ± 6.7	6.6 ± 14.8	19.9 ± 10.2	9.6 ± 5	18.6 ± 14.3	0.128
DifBW	-1.1 ± 6.7	8.2 ± 13.3	-0.075 ± 5.3	-9.6 ± 5	-0.6 ± 4.3	0.271

*FB30: G1 > G3; G4 > G2, G3, G5 (Tukey; p < 0,05)

FB30: newly formed bone 30 days after surgical procedure; FB60: newly formed bone 60 days after surgical procedure; BW30: bone marrow presence 30 days after surgical procedure; BW60: bone marrow presence 60 days after surgical procedure; Dif: diference between 30 and 60 days.

In the control group, the bone matrix still presented a disorganized pattern and was permeated by bone marrow. Although there was bone formation in the control group, it did not evolve significantly from 30 to 60 days. 

**Figure 14.**
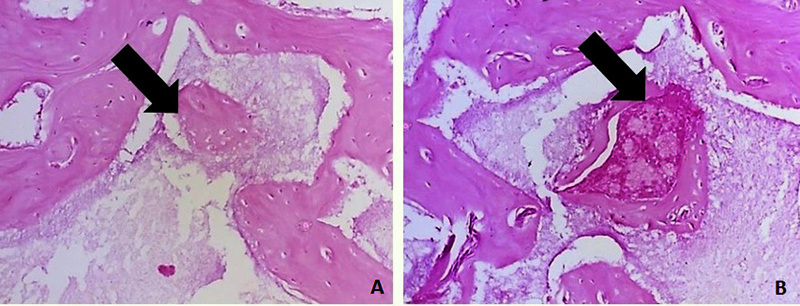
**(A)** and **(B)** Bone formation inside biphasic calcium phosphate granules at receptor site. Sections stained with hematoxylin and eosin.

**Figure 15:**
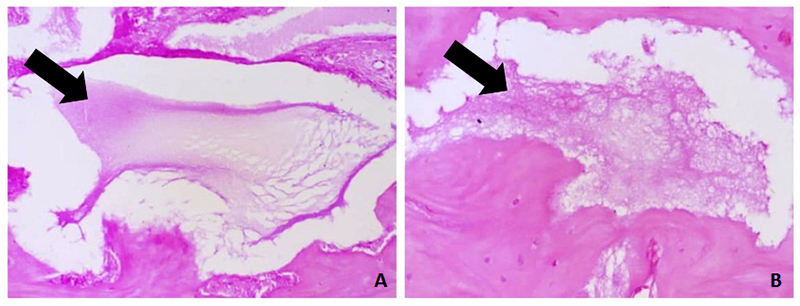
Progressive resorption of calcium phosphate granules at receptor site: **(A)** 30 and **(B)** 60 days after implantation. Sections stained with hematoxylin and eosin.

Despite the high level of bone formation around the calcium phosphate granules, no noteworthy cell proliferation was observed inside the biomaterial granules until 60 days after implantation (Fig. 14). The reabsorption of these materials was evidenced by the presence of resorption gaps and the difference in their appearance at 30 and 60 days (Fig. 15). In turn, the FBP was not visible 30 days after implantation. In the groups where phosphate and polymer were applied, with or without addition of stem cells, there was an abundant presence of highly cellularized connective tissue at 30 days.

## Discussion

The present study has evaluated, for the first time, the application and repair potential of the FBP as a biological scaffold for a biphasic calcium phosphate biomaterial associated with mesenchymal stem cells in a bone defect induced in rat femurs.

The addition of the calcium phosphate material and mesenchymal stem cells to the FBP scaffold during the surgery improved the handling and application of the association due to the formation of a moldable composite, thus reducing the time of the surgical procedure [55-58]. This was due to the malleability of the dense fibrin network formed during the stabilization of the clot [30]^.^ Previous studies applying the FBP also reported as advantages a shortening of the procedure time [38,39], easier handling and greater flexibility of the scaffold [38,37]. Gonçalves et al. [42] highlighted the stability at the receptor site provided by this scaffold. During the collection of femurs, no inflammatory infiltrates or rejection at the graft site and surrounding muscular tissue was observed, demonstrating the biocompatibility of the materials and thereby corroborating the literature [41,43,59,60].

The tomographic images indicated that total consolidation had not yet occurred after 60 days, requiring a longer period to determine the outcome of the repair process. It was verified that opacification was greater in the groups in which the biphasic calcium phosphate was applied, which was expected due to its similarity with the bone [61,62]. However, using bone density as the only parameter is not indicated as a single approach to determine the injury repair progression when calcium is applied. This approach may indicate a false overview of the recovery process.

The HU value is directly related to the density of the tissue in formation, where the higher bone density found in the treated groups may indicate tissue formation that is more compact and, therefore, more resistant [63,64]. This parameter in isolation cannot reveal whether the density was due to the presence of newly formed bone or of implanted material. In order to clarify these questions, a qualitative analysis of the defect site was required.

It is known from the literature that, after bone injury, the process of resorption is initiated, followed by proliferation and consolidation, which may or may not result in adequate remodeling [52,65]. The qualitative parameters observed in CT images indicated poor cell proliferation and remodeling in the control group, indicating that the applied associations acted as a guide for the repair process, and the application of the tested combinations improved the level of cellular migration to the lesion site [66].

The approximation of the lesion borders viewed macroscopically and by SEM from 30 to 60 days follows the expected remodeling pattern [67,43]. The characteristic granular morphology of the material was also observable [67,68]. In additional to these observations, the histological images provided a morphological overview of the tissue formation. The progressive deposition indicates the osteoconductive potential of the materials applied [59,69,70,71]. Characteristics such as regeneration from the edges of the lesion, bone formation around the applied materials and more numerous resorption gaps at 60 days are in accord with the literature [43,56,62,71-74]. The deposition of bone matrix was higher in Group 4, which received the association of FBP together with MSCs. Besides the apparent similarity in coverage between Group 4 and control group shown by SEM images, histological analysis revealed that the control group presented a less organized tissue and more infiltrated bone marrow in formed bone. In agreement with the SEM analysis, no resorption was detected inside the calcium phosphate granules in histological sections, but resorption lacunae indicate that this was occurring, but at a slow rate.

Due to the characteristic deposition of the bone tissue in formation around the calcium phosphate materials when they were implanted [75], and the linear pattern of formation in their absence, the stereological quantification [53] enabled more precise measurement of the proportion of bone formed and other constituents in the sections obtained from the defect region. The quantitative analysis corroborates that Group 4 presented greater deposition of bone matrix, in agreement with traditional sealant studies [30,45, 47,76-79].

Although the association between FBP, cells and calcium phosphate presented less bone formation than FBP and cells, the former group showed less bone-marrow infiltration in the tissue in formation, indicating the stability provided by the association between fibrin-based scaffolds and other materials [80,81]. In addition, this group presented more bone formation than the association containing only the FBP and calcium phosphate, once again showing the osteogenic improvement provided by MSC transplantation [12,30,77]. 

In the control group the bone matrix still presented a disorganized pattern and was permeated by bone marrow, indicating a lower bone density and lower resistance. In addition, there was no apparent evolution of the deposition in the bone matrix from 30 to 60 days; this finding highlights the importance of the application of bioactive materials to stimulate osteoprogenitor cell proliferation [82].

The low cell proliferation inside calcium phosphate granules indicated slow granular reabsorption. The reabsorption of the material is dependent on variables such as particle size and porosity [83], and in the case of biphasic compounds, also on the proportion of its components [55]. The porosity of this material allows vascularization, diffusion of fluids and cell migration [84] and its gradual resorption by osteoclasts [85,86]. Bose et al. [87] recommend that the ideal particle diameter be between 200 and 350 μm. In the present study, the range of calcium phosphate porosity is 10 to 100 μm, which may have impeded the diffusion of liquids and the absorption of the material in the defect produced. The resorption rate is also influenced by particle size [88,89]. In cranial defects induced in rabbits, Kon et al. [90] found that particles of 150-400 μm showed adequate resorption proportional to the speed of bone remodeling, but offered little stability compared to 1.0-2.0 mm particles. 

Thus, gradual resorption may not be totally negative where grafts need more stability and mechanical strength at the first moment of the remodeling process [62,91]. In these cases, the adding of fibrin biopolymer may improve the mechanical strength of the site, thus facilitating the incorporation of the graft into the injured area [62,72,92]. In view of these characteristics, a longer observation period is necessary to determine the efficacy of the association between FBP, calcium phosphate and MSC.

On the other hand, FBP was not visible 30 days after the implantation, which may indicate reabsorption proportional to the new bone formation. In accordance with the principle that an ideal biological framework should offer hemostasis and be degraded in the same proportion as new tissue is formed [47,93], FBP is a suitable scaffold candidate for bone repair.

We highlight that the groups in which phosphate and FBP were used, with or without addition of MSCs, presented at 30 days a greater quantity of highly cellularized connective tissue. Gasparotto et al. [30] described the ability of the FBP to capture and maintain at its site the stem cells applied, due to the dense fibrin network formed. Studies have found that the presence of fibrin increased cell proliferation and recruitment of mesenchymal stem cells to such scaffolds [94]. However, due to the unique characteristics of this FBP, further analysis is required to elucidate cell behavior in the presence of this scaffold. Nevertheless, the bioactive characteristic of this compound was validated, in addition to the advantages of being a biodegradable scaffold [77] with good capacity to integrate with biologicals [42] or synthetic biomaterials [43] that do not present toxic or cytotoxic compounds [44] and show biocompatibility with bone tissue [41].

The highly satisfactory results obtained by the association of FBP with MSCs evidence the potential clinical candidate of this association. Sixty days after implantation, a mean of 70% of observed histological sections was occupied by formed bone. In the present study, no differentiation of MSCs into osteogenic lineage was performed, indicating that this process may not be required to achieve satisfactory bone repair, which could shorten the time between the collection and application of cells and, consequently, reduce the cost of cellular expansion.

Finally, further studies applying differentiated lineage of MSCs and longer observational periods (more than 60 days) are required to reinforce the obtained results.

## Conclusions

All applied biomaterials are biocompatible and present good interaction with each other and the adjacent tissue, showing interfaces and osseointegration. Progressive bone matrix deposition was demonstrated in all treated groups, and was greater in the group that received FBP together with MSCs. FBP proved to be an excellent scaffold candidate in bone repair therapies due to its ease of application, biocompatibility, biodegradability and bioactive properties. The association has demonstrated satisfactory results, which may suggest a more effective and less costly approach for clinical applications in bone tissue engineering. Clinical trials will be necessary to prove this hypothesis.

### Abbreviations

ANVISA: Brazilian Health Regulatory Agency; BCP: biphasic calcium phosphate; DMEM: Dulbecco’s Modified Eagle Medium; FBP: fibrin biopolymer; HA: hydroxyapatite; HE: hematoxylin and eosin; MAC: minimum alveolar concentration; MSC: mesenchymal stem cell; PBS: phosphate-buffered saline; TCP: β-tricalcium phosphate.

## Supplementary material

The following online material is available for this article:

Additional file 1. Surface cell markers used on mesenchymal stem cell characterization by flow cytometry.

Additional file 2. Qualitative analysis of images obtained by computed tomography.

Additional file 3. Results of stereological analysis.

Additional file 4. Statistical analysis.
