# Supplementary material for: Fibrin biopolymer as scaffold candidate to treat bone defects in rats
Source: J Venom Anim Toxins Incl Trop Dis. 2019 Nov 4;25:e20190027. doi: 10.1590/1678-9199-JVATITD-2019-0027 (PMC6830407; doi:10.1590/1678-9199-JVATITD-2019-0027)
Supplement: Additional file 1. [file 1678-9199-jvatitd-25-e20190027-s1.pdf]

Supplementary Material to "Fibrin biopolymer as scaffold candidate to treat bone defects in rats"

Additional file 1 – Surface cell markers used on mesenchymal stem cell characterization by flow cytometry.

MARKER	EXPRESSION	TRADEMARK	REF.CODE	DILUTION	FLUOROCHROME
CD 45	Negative	ABCAM	AB123522	1/100	FITC
CD 11b	Negative	ABCAM	AB33816	1/100	PE
MHC- II	Negative	ABCAM	AB111825	1/100	PE
RT1- Aw2	Negative	ABCAM	AB119770	1/100	FITC
CD 90	Positive	ABCAM	AB226	1/100	FITC
ICAM1	Positive	ABCAM	AB23835	1/100	FITC
