# Supplementary material for: Fibrin biopolymer as scaffold candidate to treat bone defects in rats
Source: J Venom Anim Toxins Incl Trop Dis. 2019 Nov 4;25:e20190027. doi: 10.1590/1678-9199-JVATITD-2019-0027 (PMC6830407; doi:10.1590/1678-9199-JVATITD-2019-0027)
Supplement: Additional file 2. [file 1678-9199-jvatitd-25-e20190027-s2.pdf]

J Venom Anim Toxins incl Trop Dis, 2019, 25.

Supplementary Material to “Fibrin biopolymer as scaffold candidate to treat bone defects in rats”

Additional file 2 – Qualitative analysis of images obtained by computed tomography

30 days					
	**Group 1**	**Group 2**	**Group 3**	**Group 4**	**Group 5**
**Proliferation**	Absent	Discreet bone proliferation with irregular surface	Discreet	Discreet with bone proliferation of lamellar aspect	Discreet bone proliferation with irregular surface
**Remodeling**	Discreet remodeling with irregular surface	Discreet remodeling with irregular surface	Remodeling with irregular surface	Cortical on remodeling phase. Presence of mineralized thin connection	Remodeling with irregular surface

60 days					
	**Group 1**	**Group 2**	**Group 3**	**Group 4**	**Group 5**
**Proliferation**	Area between the edges of the defect	Discreet with irregular surface	Discreet with irregular surface	Exuberant bone proliferation with regular surface	Discreet formation with irregular surface
**Remodeling**	Cortical misalignment, visualization of the defect, discreet area of density and spacing of borders	In remodeling process, irregular surface and non-visualization of full defect	In remodeling process, irregular surface, discreet area with density slightly smaller than the rest of the cortical	In remodeling process, non-visualization of full defect	In remodeling process, visualization of defect, but with medullar filling
