# Supplementary material for: Fibrin biopolymer as scaffold candidate to treat bone defects in rats
Source: J Venom Anim Toxins Incl Trop Dis. 2019 Nov 4;25:e20190027. doi: 10.1590/1678-9199-JVATITD-2019-0027 (PMC6830407; doi:10.1590/1678-9199-JVATITD-2019-0027)
Supplement: Additional file 3. [file 1678-9199-jvatitd-25-e20190027-s3.pdf]

Supplementary Material to "Fibrin biopolymer as scaffold candidate to treat bone defects in rats"

Additional file 3. Results of stereological analysis.

30 DAYS						
**Group-Animal**	**FB**	**BL**	**RL**	**CT**	**BW**	**rel. tot.**
**G1-1**	63.0%	0.0%	0.0%	0.0%	37.0%	100%
**G1-2**	51.0%	0.0%	0.0%	0.0%	49.0%	100%
**G1-3**	52.8%	0.0%	0.0%	0.0%	47.2%	100%
**G1-4**	59.4%	0.0%	0.0%	0.0%	40.6%	100%
**MEAN**	56.5%	0.0%	0.0%	0.0%	43.5%	100%
**G2-1**	39.7%	52.9%	0.0%	0.0%	7.4%	100%
**G2-2**	32.0%	32.8%	1.6%	0.0%	33.6%	100%
**G2-3**	42.7%	31.3%	6.1%	0.0%	19.8%	100%
**G2-4**	40.7%	33.0%	5.9%	0.0%	20.3%	100%
**MEAN**	38.8%	37.5%	3.4%	0.0%	20.3%	100%
**G3-1**	22.0%	27.2%	14.4%	33.3%	3.0%	100%
**G3-2**	32.6%	20.4%	2.3%	34.1%	10.6%	100%
**G3-3**	26.5%	21.6%	8.1%	36.3%	7.1%	100%
**G3-4**	32.6%	24.2%	3.8%	28.8%	10.6%	100%
**MEAN**	28.4%	23.4%	7.1%	33.1%	7.8%	100%
**G4-1**	67.0%	0.0%	0.0%	0.0%	33.0%	100%
**G4-2**	66.9%	0.0%	0.0%	0.0%	33.1%	100%
**G4-3**	68.3%	0.0%	0.0%	0.0%	31.7%	100%
**G4-4**	59.1%	0.0%	0.0%	0.0%	40.9%	100%
**MEAN**	65.3%	0.0%	0.0%	0.0%	34.7%	100%
**G5-1**	19.8%	28.1%	30.2%	21.8%	0.0%	100%
**G5-2**	36.1%	29.2%	15.5%	13.7%	5.6%	100%
**G5-3**	54.3%	21.7%	20.2%	3.8%	0.0%	100%
**G5-4**	48.9%	26.0%	14.5%	6.9%	3.8%	100%
**MEAN**	39.8%	26.2%	20.1%	11.5%	2.3%	100%

FB: newly formed bone. **BL**: calcium phosphate biomaterial. **RL**: resorption lacunae. **CT**: cellular connective tissue. **BW**: bone marrow. **Rel. tot:** relative total.

60 DAYS						
**Group-Animal**	**FB**	**BL**	**RL**	**CT**	**BW**	**rel. tot.**
**G1-1**	68%	0%	0%	0%	32%	100%
**G1-2**	42.0%	0.0%	0.0%	0.0%	58.0%	100%
**G1-3**	57.0%	0.0%	0.0%	0.0%	43.0%	100%
**G1-4**	63.7%	0.0%	0.0%	0.0%	36.3%	100%
**MEAN**	57.6%	0.0%	0.0%	0.0%	42.4%	100%
**G2-1**	52.0%	16.7%	21.3%	0.0%	10.0%	100%
**G2-2**	48.6%	36.4%	7.9%	0.0%	7.1%	100%
**G2-3**	27.3%	42.4%	9.8%	0.0%	20.5%	100%
**G2-4**	53.8%	22.7%	12.9%	0.0%	10.6%	100%
**MEAN**	45.4%	29.6%	13.0%	0.0%	12.0%	100%
**G3-1**	56.8%	16.4%	12.4%	4.0%	10.4%	100%
**G3-2**	47.6%	19.0%	25.5%	2.0%	5.9%	100%
**G3-3**	45.1%	26.6%	21.3%	0.0%	6.9%	100%
**G3-4**	44.1%	27.7%	18.4%	2.0%	7.8%	100%
**MEAN**	48.4%	22.4%	19.4%	2.0%	7.8%	100%
**G4-1**	71.4%	0.0%	0.0%	0.0%	28.6%	100%
**G4-2**	78.1%	0.0%	0.0%	0.0%	21.9%	100%
**G4-3**	75.5%	0.0%	0.0%	0.0%	24.5%	100%
**G4-4**	75.0%	0.0%	0.0%	0.0%	25.0%	100%
**MEAN**	75.0%	0.0%	0.0%	0.0%	25.0%	100%
**G5-1**	50.4%	32.2%	16.0%	0.0%	1.4%	100%
**G5-2**	63.5%	22.8%	12.8%	0.0%	0.8%	100%
**G5-3**	53.0%	31.3%	11.1%	0.0%	4.5%	100%
**G5-4**	66.6%	18.4%	15.1%	0.0%	0.0%	100%
**MEAN**	58.4%	26.2%	13.7%	0.0%	1.7%	100%

FB: newly formed bone. **BL**: calcium phosphate biomaterial. **RL**: resorption lacunae. **CT**: cellular connective tissue. **BW**: bone marrow. **Rel. tot:** relative total.
