# Supplementary material for: Fibrin biopolymer as scaffold candidate to treat bone defects in rats
Source: J Venom Anim Toxins Incl Trop Dis. 2019 Nov 4;25:e20190027. doi: 10.1590/1678-9199-JVATITD-2019-0027 (PMC6830407; doi:10.1590/1678-9199-JVATITD-2019-0027)
Supplement: Additional file 4. [file 1678-9199-jvatitd-25-e20190027-s4.pdf]

Supplementary Material to "Fibrin biopolymer as scaffold candidate to treat bone defects in rats"

Additional file 4. Statistical analysis

Group		FB 30	BW 30	FB 60	BW 60	DIFFB	DIFBW
**1**	Mean	56.550	43.450	57.675	42.325	1.125	-1.125
	Std. Deviation	5.6152	5.6152	11.3881	11.3881	6.7594	6.7594
**2**	Mean	38.775	20.275	45.425	12.050	6.650	-8.225
	Std. Deviation	4.6857	10.7012	12.2742	5.8370	14.8181	13.3290
**3**	Mean	28.425	7.825	48.400	7.750	19.975	-.075
	Std. Deviation	5.1591	3.6151	5.7902	1.9296	10.2996	5.3137
**4**	Mean	65.325	34.675	75.000	25.000	9.675	-9.675
	Std. Deviation	4.1987	4.1987	2.7580	2.7580	5.0009	5.0009
**5**	Mean	39.775	2.350	58.375	1.675	18.600	-.675
	Std. Deviation	15.3487	2.8113	7.8826	1.9687	14.3557	4.3919

FB30: newly formed bone 30 days after surgical procedure; **FB60:** newly formed bone 60 days after surgical procedure; **BW30:** bone marrow presence 30 days after surgical procedure; **BW60:** bone marrow presence 60 days after surgical procedure; **Dif**: difference between 30 and 60 days.
